# Comment on “Expert evaluation of Generative Pre-trained Transformer-4o and Gemini responses to patient questions on carotid endarterectomy”

**DOI:** 10.1590/1806-9282.20260619

**Published:** 2026-08-10

**Authors:** Huseyin Durmaz, Semiha Durmaz

**Affiliations:** 1 Konya City Hospital, Department of Cardiovascular Surgery – Konya, Türkiye.; 2 Konya City Hospital, Department of Internal Medicine – Konya, Türkiye.

Dear Editor,

I have read with interest the article entitled “Expert evaluation of Generative Pre-trained Transformer-4o and Gemini responses to patient questions on carotid endarterectomy”^1^ recently published in Revista da Associação Médica Brasileira by Dr. Rahman et al. The study is an important and timely research given the increasing role of artificial ıntelligence-based tools in health communication and patient education.

The data presented in the study deserve commendation for their use of a blinded expert panel and a structured evaluation approach. However, I believe that some methodological and interpretive aspects of the study require further evaluation.

Firstly, while the process of creating the question set is comprehensively described, a standardized and reproducible methodology is not presented. The use of heterogeneous sources such as search engines, social media, and forums increases the risk of selection bias. Unsystematic and insufficiently detailed search strategies can significantly limit the reproducibility of studies, leading to weakened methodological reliability. Furthermore, it is emphasized that the use of systematic and transparent methods reduces bias in research and increases the reliability of findings^2^. Therefore, a more systematic and reproducible question-formulating approach will strengthen the validity and generalizability of the findings.

Secondly, the evaluation is based on a limited expert panel consisting of only four cardiovascular surgeons, and the lack of reporting of inter-rater agreement is a significant methodological deficiency. Current methodological studies emphasize that in Likert-scale evaluations performed by multiple raters, reporting of inter-rater agreement analyses is necessary to ensure the reliability of measurements and the interpretability of results^3^.

In conclusion, the study by Dr. Rahman et al. presents valuable and noteworthy findings regarding the use of Large Language Model in the field of carotid endarterectomy. I believe that further research in this area will make significant contributions to the literature, and I hope that the authors will consider these suggestions in their future studies.

Thank you for giving me the opportunity to share my thoughts on this important study.

## Data Availability

The datasets generated and/or analyzed during the current study are available from the corresponding author upon reasonable request.
